# A Different Way to Stay in Touch with ‘Urban Nature’: The Perceived Restorative Qualities of Botanical Gardens

**DOI:** 10.3389/fpsyg.2017.00914

**Published:** 2017-05-31

**Authors:** Giuseppe Carrus, Massimiliano Scopelliti, Angelo Panno, Raffaele Lafortezza, Giuseppe Colangelo, Sabine Pirchio, Francesco Ferrini, Fabio Salbitano, Mariagrazia Agrimi, Luigi Portoghesi, Paolo Semenzato, Giovanni Sanesi

**Affiliations:** ^1^Experimental Psychology Laboratory, Department of Education, Roma Tre UniversityRome, Italy; ^2^Department of Human Studies, Libera Università Maria SS. AssuntaRome, Italy; ^3^Department of Agricultural and Environmental Science, University of BariBari, Italy; ^4^Department of Dynamic and Clinical Psychology, Sapienza University of RomeRome, Italy; ^5^Department of Agrifood Production and Environmental Sciences, University of FlorenceFlorence, Italy; ^6^Department of Agriculture, Food and Forest Systems Management, University of FlorenceFlorence, Italy; ^7^Department for Innovation in Biological, Agro-food and Forest Systems, Tuscia UniversityViterbo, Italy; ^8^Department of Land, Environment, Agriculture and Forestry, University of PaduaPadua, Italy

**Keywords:** botanical garden, perceived restorativeness, subjective well-being, psychological benefits, physical benefits

## Abstract

Botanical gardens represent interesting arenas for research in environmental psychology and environment-behavior relations. They can be considered a very particular type of restorative environment and also have a relevant social function for the promotion of a more sustainable lifestyle in current societies. In this paper, we present a study assessing the relationship between the perceived restorativeness, the psychological and physical benefits experienced, and the subjective well-being reported by visitors of botanical gardens in four different cities in Italy (*N* = 127). As expected, a bootstrapping mediation model supported the idea that perceived restorativeness of botanical gardens significantly predicts visitors’ subjective well-being, both directly and indirectly through perceived physical and psychological benefits of the visit. A moderation model also revealed that the relationship between restorativeness and well-being varies across respondents with different socio-demographic characteristics, being stronger for singles as compared to couples with and without children, respectively. The theoretical and practical implications of these findings are discussed.

## Introduction

A botanical garden or arboretum is a space for cultivating, collecting, and studying plants. These green spaces usually contain plant collections organized in different ways (geographical origin, bioma, landscape, taxa, functions, etc.). The origin of botanical gardens is rooted in the medieval age, when monks cultivated medicinal plants in open spaces that were called physic gardens. Nowadays, botanical gardens not only collect and display a wide variety of plant species for scientific research and nature conservation purposes, but also provide visitors and community members with such opportunities as guided tours, temporary exhibitions, and educational activities. Botanical gardens often are managed by scientific research institutes such as in universities, by municipalities, or by other public or private institutions.

In the context of the urban green infrastructure and ecosystems, botanical gardens are a patch of the urban forest and contribute to the formation of the whole green infrastructure. Over the years, botanical gardens have been in the study focus of forestry, agriculture, botany, and horticultural science. Furthermore, given the increasing “urbanization” of contemporary human habitats, botanical gardens have assumed also a strong social relevance. According to recent estimates by the UN Department of Economic and Social Affairs, the world urban population was approximately 54% in 2014, and is projected to rise to 66% by 2050^1^. Within urban settings and dense metropolitan areas, botanical gardens provide spaces in which the general public has the opportunity of staying in touch with natural elements, and to take refuge from urban stressors such as crowding, noise, air pollution, and heat. Botanical gardens are also characterized by a high level of biodiversity, richness, and species variety, which is less common in other more “standard” urban green spaces, such as urban forests, parks, and playgrounds. Recently, the role of biodiversity of green areas in promoting higher levels of well-being has gained empirical support (Fuller et al., 2007; Carrus et al., 2015; Sandifer et al., 2015). As such, botanical gardens could be considered as a very special type of “restorative environment” (see Hartig, 2004), and represent interesting arenas in which to conduct research on environment-behavior relations, and, in particular, for research on the interplay between environmental psychology and urban forestry.

### Botanical Gardens As Restorative Environments

Restorative environments have been defined as settings capable to “promote (rather than merely permit)” the recovery of those mental resources used by the individual to face daily life tasks, which usually is associated with positive outcomes, such as renewal of cognitive functions, stress reduction, increase in positive emotions, and psychological well-being (Hartig, 2004, p. 274). Empirical research on restorative environments has consistently shown the benefits of human-nature transactions in several domains over many decades. Humans are usually positively attracted to natural environments (e.g., Van den Berg et al., 2007; Carrus et al., 2013), so that staying in touch with wilderness, nature, and trees allows people to gain some distance from everyday life, reflect on their goals from a different perspective, and feel relaxed and at peace (e.g., Kaplan and Talbot, 1983). In addition, various authors have suggested that the experience of natural settings can have positive consequences for human cognition and health, and this idea has been supported extensively by empirical research over the last four decades, with evidence coming from different perspectives and approaches (e.g., Ulrich, 1984; Berman et al., 2008; Mitchell and Popham, 2008; Hartig et al., 2011).

Studies on restorative environments traditionally assumed an evolutionary “Biophilia” perspective (e.g., Wilson, 1984) as a general theoretical framework, suggesting that human beings have developed an innate tendency to respond positively to nature as a consequence of evolutionary adaptation processes. This positive response also includes psychological restoration, as conceived by such authors as Ulrich (1984), or Kaplan and Kaplan (1989), in terms of stress reduction (Stress Reduction Theory – SRT) and recovery of directed attention (Attention Restoration Theory – ART), respectively. Many empirical studies have offered support for both SRT (e.g., Hartig et al., 1991; Ulrich et al., 1991) and ART (e.g., Staats et al., 2003; Berto, 2005), and theoretical frameworks have been proposed to understand better the connections between the two (see Kaplan, 1995).

In particular, ART identifies four basic properties of settings to promote psychological restoration among its users or viewers: being-away, which implies a change of scenery and/or experience from daily routines; fascination, which refers to the capability of esthetically pleasant environments to catch one’s attention without mental effort; extent, which refers to the properties of connectedness and scope in an environment in which all elements are coherently related to one another, as well as the promise to engage one’s mind through more than that which is immediately perceived; and compatibility, which has to do with the level of perceived congruence between the characteristics of the environment and people’s needs and inclinations. To measure these properties, Hartig et al. (1997) developed the Perceived Restorativeness Scale (PRS), consisting of 26 items measuring the four restorative properties proposed by ART. Through the use of this and other similar tools, and concurrent measures of physiological and cognitive restoration, environmental psychological studies have consistently found empirical support for the assumption that people respond more positively to natural vs. built settings (Hartig et al., 2003; Staats et al., 2003; Van den Berg et al., 2003; Berto, 2005), and that the perception of the restorative qualities of a setting is positively associated with the perception of its “naturalness” (e.g., Carrus et al., 2013, 2015). In addition, links between psychological and physical well-being have emerged in several studies on the experience of nature (Bodin and Hartig, 2003; Hansmann et al., 2007; Pressman et al., 2009). Taken together, this body of literature would lead us to expect that perceived restorativeness can be a predictor of people’s subjective well-being when visiting natural settings. At the same time, we could argue that this relationship is explained by people’s awareness of the benefits (either physical, psychological, or both) that one receives when interacting with nature and restorative environments in general. Indeed, there are previous studies offering empirical support for the assumption that the perceived restorative qualities of urban and peri-urban natural settings could be a direct predictor of subjective well-being for people who visit them (see, for example, Carrus et al., 2015), and also for the idea that perceived physical and psychological benefits are mechanisms mediating the restorative effects of visiting urban natural spaces (see, for example, Lafortezza et al., 2009). To our knowledge, however, the mediating role of perceived benefits on the relationship between perceived restorativeness and subjective well-being derived from visiting urban natural settings has not been tested formally in a unique model.

Recent directions in the study of the benefits associated with nature experience have tried to understand better the role of some personal characteristics in moderating the effects of contact with nature. In this regard, some studies have outlined differences by age group in preference, perceived restoration, and positive outcomes as related to contact with natural environments, while the effect of gender is often non-significant (Scopelliti and Giuliani, 2004; Regan and Horn, 2005; Barton and Pretty, 2010). In addition, social interaction when experiencing green environments can decrease the restorative potential of contact with nature, because other people may represent a factor preventing a complete perception of the positive qualities of the environment (Scopelliti and Giuliani, 2004, 2006; Staats and Hartig, 2004; Carrus et al., 2015; Scopelliti et al., 2016). In particular, having children has been associated in the literature with lower levels of well-being for parents, and women above all, because of an increase of family demands and distress, and a decrease in social support that partners receive from each other (Ross et al., 1990). As a consequence, it is possible to hypothesize that increase in the complexity of one’s household composition (namely going from being single, to living with a partner, to living with a partner and children) would lead to different ways of spending time in natural environments, thus affecting the perception of the restorative components of natural environments. In fact, families function on different levels, and according to classical ecological models of human development, family status and relationships are assumed to directly and indirectly influence people’s psychological states and well-being (e.g., Bronfenbrenner, 1986). In situations in which individuals face challenges, their partner and family members can be important resources for overcoming problems and coping with stressful experiences (Thoits, 1995; Friborg et al., 2003). At the same time, family issues or family demands can represent a burden that does not allow for a complete restorative experience in nature, thus reducing the “pure” effects of perceived restorativeness of a setting on the well-being of individuals (Patterson, 2002). Therefore, we can argue that people with different household compositions will have different experiences of public green areas such as botanical gardens, and also display differences in assessing the restorative function of these particular green spaces. However, the literature on the role of these variables is still scant and fragmented, and further research is undoubtedly needed.

In sum, we argue here that botanical gardens do fit well with the definition of restorative environments. In fact, botanical gardens are characterized by a high concentration of plant species and natural elements in a relatively wide space within the urban environment. Also, they are places in which visitors can find opportunities for staying away from everyday routines, for being attracted by pleasant environmental stimuli linked in a coherent fashion, and for fulfilling relaxation, escape, and contemplation needs. Moreover, they are suitable environments to analyze the role of possible moderators of the positive outcomes of users’ experiences in visiting natural places.

In addition to their potential for offering psychological restoration, botanical gardens can have a relevant function for the promotion of a more sustainable lifestyle in contemporary urbanized societies (e.g., Ballantyne et al., 2008). Botanical gardens are, in fact, not only useful tools for directly conducting conservation studies or climate change research (e.g., Pinheiro et al., 2006; Primack and Miller-Rushing, 2009), but also can be considered as strong means of environmental information for the general public. Being visited by large numbers of people, botanical gardens can provide information on the health benefits of contact with nature, on plants and ecosystem conservation issues, as well as on environmental issues in general, such as climate change or global warming. Understanding how people perceive these settings and the benefits they might get from visiting them, therefore, could help in promoting more frequent and longer visits by the public (Waylen, 2006). This promotion, in turn, could help in fostering a more balanced view of human-nature relations among residents of urban and peri-urban areas, and help to address the crucial challenge of promoting a transition to more sustainable lifestyles in current densely urbanized societies.

Despite the presence of a large number of botanical gardens in different cultural and geographical contexts of the world, studies considering these settings as restorative environments still are lacking. Some exceptions focusing on the experience of visiting botanical gardens are worth noting (e.g., Steinhauer et al., 2007; Mattson, 2010; Ward et al., 2010; Lückmann et al., 2013). However, these studies are mostly descriptive, and they focus on who the visitors are and what they do, with no deep analysis of what leads to positive outcomes for individuals who visit botanical gardens. In particular, while the existing studies seem to converge in suggesting that the experience of visiting botanical gardens could be restorative in many ways, stronger evidence is still needed to ascertain whether people perceive botanical gardens as restorative environments. Also, it would be important to understand to what extent this perception can be generalized across different socio-demographic categories, and whether the perceived restorativeness of botanical gardens is positively linked to subjective well-being.

### The Present Study

In this paper, we present a study that assessed relationships among the perceived restorative properties, the psychological and physical benefits experienced, and the subjective well-being reported by visitors of botanical gardens located in four different cities in Italy (Bari, in the south, Rome and Florence, in the center, and Padua, in the north). We tested a mediation model in which the perceived restorativeness of botanical gardens was set as a predictor of subjective well-being, both directly and indirectly, through perceived physical and psychological benefits. We also tested a moderation model where the relationship between perceived restorativeness and subjective well-being was assessed across respondents with different household compositions.

The aims and hypotheses of this study are:

(i) To assess the perceived restorativeness of botanical gardens, and to further explore the links among perceived restorativeness, perceived psychological and physical benefits, and self-reported well-being, as basic psychological mechanisms involved in the connection of nature and psychological restoration. As with previous studies, we expect a positive link between perceived restorativeness and well-being, both directly and indirectly, through self-reported physical and psychological benefits (Lafortezza et al., 2009; Carrus et al., 2015).
(ii) To understand whether the relationship between perceived restorativeness and self-reported well-being varies across participants as a function of different socio-demographic characteristics, such as gender, age, or household composition.

## Materials and Methods

### Study Site Selection

**FIGURE 1:**
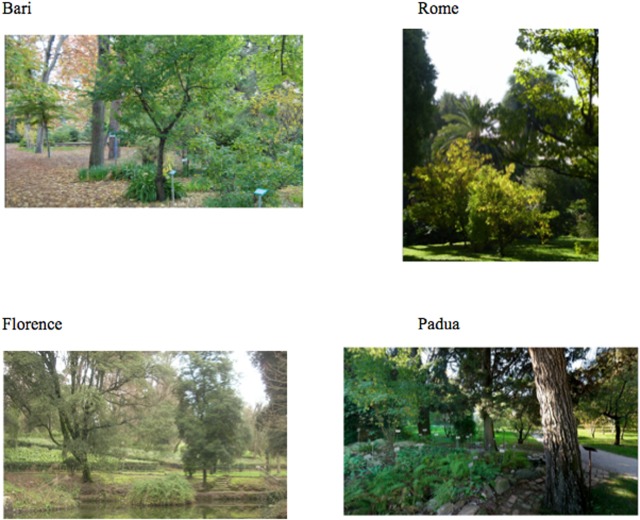
Sample images of the selected botanical gardens in Bari, Rome, Florence, and Padua.

Four different botanical gardens were selected for the purpose of this study, located in cities along a south-north geographic gradient in Italy: Bari (in the southern part of the country), Rome and Florence (in the center), and Padua (in the north). Sample images of the selected sites are provided in **Figure 1**.

The size of the botanical gardens considered varied from 1 hectare (Bari), to 2.2 hectares (Padua) and 2.4 hectares (Florence), up to 12 hectares (Rome). Although the Rome garden is much bigger than the other three sites, no significant differences in the main outcome variable of our study (i.e., well-being after the visit) were detected across the four sites.

### Participants

A convenience sample of 127 respondents visiting Rome, Bari, Padua, and Florence botanical gardens participated in the study (Age range 19–86; Mean age = 40.69; *SD* = 16.83; 50% women). Participants were 28 subjects for the Rome site, 25 subjects for the Bari site, 24 subjects for the Padua site, and 50 subjects for the Florence site. Subjects were approached by trained interviewers (undergraduate students of psychology and forestry science programs) on site while spending their leisure time at the different locations, and were invited to take part in the study by completing a 10-min paper-and-pencil questionnaire. Participants were informed about the anonymous character of the survey, and assured that their answers would be used only for scientific research purposes and aggregated for statistical analyses. Participants expressed their oral informed consent and voluntarily agreed to participate in the study.

### Procedure and Measures

The questionnaire was organized into different sections. Section 1 consisted of open-ended, multiple-choice, and Likert-type questions on different aspects of the visit (e.g., length and frequency of visits, main activity performed during the visit), plus socio-demographic data (e.g., gender, age, household composition; in particular, this variable asked to participants to indicate whether their family status was single, couple without children, or couple with children, although it was not recorded directly whether participants actually were visiting the garden alone, with a partner, or with children). Section 2 contained eight items from the Italian version of Hartig et al.’s (1997) PRS (Pasini et al., 2009), measuring the perceived restorative properties of the settings (score range 0–4; Cronbach’s alpha = 0.73). Section 3 contained two items measuring the physical and psychological benefits perceived during the visit (i.e., “Do you feel physical benefits from visiting this place?”; “Do you feel psychological benefits from visiting this place?”), and two items measuring the perceived well-being change after the visit (i.e., “Overall, how much did visiting this place make you feel better than before the visit?”; “Overall, how much did visiting this place make you feel better than usual?”), all measured on a 5-point scale (scores range from 0 to 4). Data from the open-ended questions and the experience-related variables are not presented in this paper. All of these measures were derived and adapted from previous studies (e.g., Lafortezza et al., 2009; Carrus et al., 2013, 2015; Scopelliti et al., 2016).

### Statistical Analysis

To investigate relationships among perceived restorativeness, physical and psychological benefits, and subjective well-being, we computed the zero-order correlations among these variables first, and then used the INDIRECT macro, which allowed us to test the role of two mediators simultaneously (namely physical and psychological benefits felt during the visit) in the relationship between perceived restorativeness and subjective well-being change after the visit. A bootstrapping procedure (with 5000 bootstrap samples) to estimate 95% confidence intervals (95% CI) was used. According to Preacher and Hayes, a 95% CI that does not include zero provides evidence of a significant indirect effect (Preacher and Hayes, 2008, for more details). The bootstrapping procedure has been suggested to represent the most trustworthy test for assessing the effects of mediating models (see Hayes and Scharkow, 2013, for a recent review). The 0.05 level of significance was adopted in all analyses.

To generalize the main relationships of this study, we explored the patterns linking various socio-demographic factors to our measures of perceived restorativeness and subjective well-being as well. No substantial direct associations were found between restorativeness, well-being, and the main socio-demographic factors (gender, age, household composition). Nevertheless, we focused on possible moderating effects of these factors. Various models that aimed to assess whether the effect of restorativeness on well-being varied as a function of the socio-demographic variables were tested. In particular, a model on the moderating effect of household composition on the relation between restorativeness and subjective well-being was tested, using the PROCESS macro (see Hayes, 2013, for more details). In this way, we assessed whether (and how) the relationship between perceived restorativeness and well-being changed as a function of the participants’ household composition (e.g., singles, couples with no children, couples with children). Prior to the statistical tests, we checked for possible differences in the main outcome variable of our models (i.e., well-being after the visit) as a function of either of the four different study sites and of the three levels of the moderator; no significant differences emerged (*F* < 1; *p* = 0.502 and *p* = 0.585, respectively).

## Results

The levels of perceived restorativeness (computed as the average score in the perceived restorative properties), physical and psychological benefits, and subjective well-being in visiting botanical gardens were generally high. Bivariate correlations and descriptive statistics are displayed in **Table 1**. As predicted, the findings reveal that restorativeness and well-being are significantly positively related to each other. Also, both physical and psychological benefits felt during the visit significantly are positively correlated with the perception of the restorativeness of botanical gardens. We also detected significant positive correlations between physical/psychological benefits and well-being felt after the visit.

**Table 1:** Means, standard deviations, and intercorrelations among independent variable, mediators, and outcome.

	1	2	3	4
(1) Well-being	1			
(2) Restorativeness	0.56^∗∗^	1		
(3) Psychological benefits	0.71^∗∗^	0.54^∗∗^	1	
(4) Physical benefits	0.65^∗∗^	0.48^∗∗^	0.58^∗∗^	1
M (*SD*)	2.53 (*0.76*)	2.49 (*0.51*)	2.78 (*0.86*)	2.28 (*0.93*)

*^∗∗^ p < 0.01; N = 116; All scores range on 0–4 scale. Missing values deleted listwise.*

**FIGURE 2:**
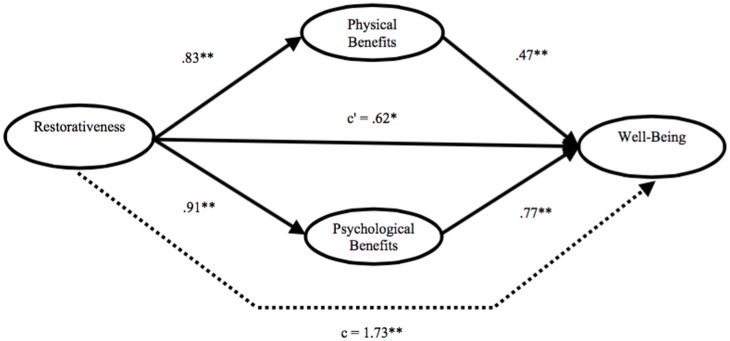
Mediating model which shows the effect of restorativeness on well-being after the visit through physical and psychological benefits felt during the visit. NOTE: Path values represent unstandardized regression coefficients. The (c^∗^) value represents the effect, from bootstrapping analyses, of the restorativeness on well-being after the mediators are included. Dotted line (c) represents the total effect of the restorativeness on well-being prior to the inclusion of the mediating variables. *^∗^p <* 0.05; *^∗∗^p <* 0.001.

As shown in **Figure 2**, the mediating model was estimated to derive the total, direct, and indirect effects of perceived restorativeness on self-reported change in well-being after the visit, through physical as well as psychological benefits felt during the visit. Mediation analyses revealed a simultaneous significant positive indirect effect of restorativeness on well-being through both the two mediators hypothesized: i.e., (a) physical (point estimate = 0.39, 95% percentile CI = 0.21–0.61) and (b) psychological (point estimate = 0.71, 95% percentile CI = 0.40–1.03) benefits felt during the visit.

**FIGURE 3:**
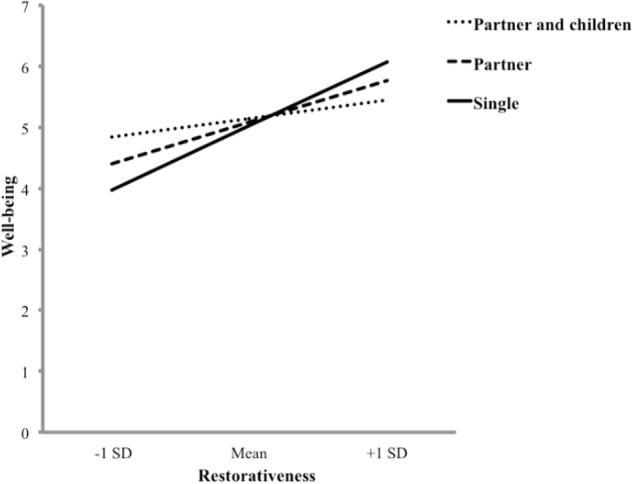
Moderation of the effect of restorativeness on well-being by household composition.

**Figure 3** shows the moderation model tested to derive the conditional effect of perceived restorativeness on well-being, as a function of variations in household composition. We found a significant positive main effect of perceived restorativeness on well-being (*t* = 6.46; *p* < 0.001), while no significant main effect of household composition on well-being was detected (*p* > 0.1). However, an interaction effect among perceived restorativeness and household composition on well-being was detected (*t* = 3.45; *p* < 0.01). Conditional effects (i.e., “simple slopes”; see Hayes, 2013) of perceived restorativeness on well-being were estimated using the “pick-a-point” approach, with the sample mean and plus and minus one standard deviation from the mean representing the categories of “unmarried/married partner,” “single,” and “partner and children” household composition, respectively. Perceived restorativeness was significantly and positively related to well-being at two points (*p* < 0.05; conditional effects were 2.27 at “single,” and 1.45 at “unmarried/married partner” status, respectively), with the effect approaching zero when household composition included children.

## Discussion And Concluding Remarks

This paper provides empirical support for the role of botanical gardens as restorative environments. The levels of perceived restorativeness of botanical gardens, physical and psychological benefits experienced during the visit, and overall individual well-being, were high. This adds further evidence to the positive outcomes of spending time within such a natural setting (Steinhauer et al., 2007; Ward et al., 2010), and, more generally, within natural environments that are rich in biodiversity (Fuller et al., 2007; Carrus et al., 2015; Sandifer et al., 2015). These results are also in line with previous literature on the benefits of contact with nature in general, be they physical (Bodin and Hartig, 2003; Bowler et al., 2010; Thompson Coon et al., 2011) or psychological (Ulrich et al., 1991; Hartig et al., 1996; White et al., 2013; Scopelliti et al., 2016).

In addition, some key mechanisms promoting individual well-being were identified in this work. As expected, our data reveal that the experience of botanical gardens leads people to perceive the restorative properties of this particular environment, which fosters individual well-being through the increase of perceived physical and psychological benefits.

As a general speculation, the results that emerged in our study are line with previous research outlining the role of cognitive and affective processes in triggering well-being, including the perception of specific features of the environment and the emotions associated with such perceptions (Abraham et al., 2010; Grahn and Stigsdotter, 2010; Scopelliti et al., 2016). More specifically, our findings might be consistent with the assumption that perceiving the restorative properties of nature can be a prerequisite for awareness and recognition of the beneficial outcomes of spending personal time in nature, particularly within dense urban settings (see Carrus et al., 2015).

We might also speculate that when people are experiencing mental states related to increased awareness, such as in the case of mindfulness practice and meditation, the link between perceived restorativeness and well-being should be stronger. Indeed, recent studies have explored the links between mindfulness, restoration, and connectedness to nature, although with mixed results (e.g., Barbaro and Pickett, 2016; Lymeus et al., 2017). Thus, these aspects are worthy of further investigation in future research. Our findings also suggest that being aware of these benefits, both at the physical and psychological level, can, in turn, lead to subjective well-being after experiencing restorative environments, as suggested in an earlier study by Lafortezza et al. (2009), in which psychological and physical benefits were aggregated into a single indicator. Here, we could shed more light on those previous findings, as we estimated the weight of psychological vs. physical benefits separately. From our findings, it appears that the psychological, rather than the physical benefits have a greater mediating role, and again, this could be an issue for further investigation in future studies.

Finally, the role of potential moderators in the relationship between perceived restorativeness and well-being was considered, thus contributing to a still scant literature. While no significant difference with respect to gender and age emerged, an interesting role of household composition was found. The relationship between perceived restorativeness and well-being was stronger for singles and decreased for visitors with a partner, with the effect approaching zero when household composition included children. This finding is consistent with previous research on restorative environments. For instance, Staats and Hartig (2004) analyzed the role of social interaction in the restorative experience, and found that the presence of other people may diminish the benefits in natural environments, when perceived safety is controlled. Likewise, Scopelliti and Giuliani (2006) considered social interaction as a potential moderator of positive outcomes for the elderly. They found an effect of social interaction on the restorative potential of natural environments, which was perceived to be higher when people were alone. The authors suggest that social interaction in natural environments may represent a source of distraction from the relationship with the environment, which is restorative in itself. Similar detrimental effects of social interaction on the positive outcomes of contact with nature were also found by Carrus et al. (2015).

It is important to clarify that the household composition factor in the present study refers to the actual family status of the respondents, rather than whether family members were present or not during the visit. Nevertheless, even if family members might have not been present in some cases, we shall recall that family status is a condition that influences peoples’ psychological states independently of the actual presence of family members (e.g., Bronfenbrenner, 1986). In fact, families are relational systems fulfilling several functions on different levels, allowing the individual development, socialization, and health of their members. In situations in which individuals face challenges, social support is an important resource for overcoming problems, pursuing goals, and facing stressful experiences (Thoits, 1995), so that the partner and family members can help in promoting higher levels of well-being and health together with external social support sources, constituting an important part of individuals’ resilience (Friborg et al., 2003). However, family members’ issues or problems may be the origin of stressful experiences, being risk factors for stress related health problems (Patterson, 2002).

We might speculate more generally that family demands, while being a source of positive outcomes and satisfaction in many areas of human life, is also a burden that does not allow for a complete restorative experience in nature, thus reducing the “pure” effects of the perceived restorativeness of a setting on well-being. This is also consistent with several research findings that suggest a link between family demands and poorer health conditions, especially for women (Ross et al., 1990; Artazcoz et al., 2001, 2004). In the case of our study, we could for example speculate that participants with children have associated the botanical garden with family trips involving the children, and associated demands, whereas for those without children the visit to the botanical garden has more escape-related associations. This is an issue that would deserve further investigation in future studies.

However, some limits of our studies should be also acknowledged. In fact, although our data were gathered in the context of a larger national research project in which many different sites have been studied (see Carrus et al., 2015), because of the cross-sectional nature of our study, we do not have a direct comparison between botanical gardens and other settings. Therefore, future research should aim at clarifying better whether visiting botanical gardens actually promotes well-being more than visiting other types of restorative environments, either through field experiments or longitudinal datasets. Also, it will be interesting to link more the actual characteristics of the botanical gardens and the specific features of the visit to restoration outcomes. These aspects, and the role of other potential moderators of restorative nature experience, would benefit from further and deeper investigation in future research.

More generally, our findings suggest that the experience of botanical gardens is something that should be promoted in densely urbanized settings in which the majority of people in the world currently live. Not only, as we showed, can visiting botanical gardens can be a source of positive outcomes and well-being for urban residents, but also, as we argued, botanical gardens could be a powerful tool for promoting a better and more balanced relationship with nature in cities among urban residents, thus contributing to the desire for a transition to more sustainable lifestyles in the society-at-large.

## Ethics Statement

Participants to this study expressed their oral consent to take part to the survey, and voluntary accepted to fill in an anonymous self-reported paper and pencil questionnaire. This study did not require approval from an ethical committee according to Italian regulations.

## Author Contributions

GCa, MS, RL, GS, and SP shared the task of writing and revising the main text. AP run the statistical tests and wrote the results section. GCo, FF, FS, MA, LP, and PS coordinated the data collection in the different study sites, and contributed to text revision.

## Conflict of Interest Statement

The authors declare that the research was conducted in the absence of any commercial or financial relationships that could be construed as a potential conflict of interest.

## Footnotes

1 http://www.un.org/en/development/desa/news/population/world-urbanization-prospects-2014.html
